# Symptomatic hyponatremia following lateral medullary infarction: a case report

**DOI:** 10.1186/1471-2377-14-111

**Published:** 2014-05-22

**Authors:** Jeong-Min Kim, Kwang-Yeol Park, Do Hyoung Kim, Jae-Han Bae, Dong-Woo Shin, Young Chul Youn, Oh-Sang Kwon

**Affiliations:** 1 Department of Neurology, Chung-Ang University Hospital, Chung-Ang University College of Medicine, Seoul, South Korea; 2 Department of Internal Medicine, Chung-Ang University Hospital, Chung-Ang University College of Medicine, Seoul, South Korea

**Keywords:** Hyponatremia, Syndrome of inappropriate secretion of antidiuretic hormone, Cerebral salt wasting syndrome, Lateral medulla

## Abstract

**Background:**

Hyponatremia has been reported from patients with severe neurological disease, and the syndrome of inappropriate secretion of antidiuretic hormone and cerebral salt wasting syndrome are the two main etiologies of hyponatremia after brain injury. Here we describe a patient with a lateral medullary infarction who experienced symptomatic hyponatremia with finding suggestive of syndrome of inappropriate secretion of antidiuretic hormone followed by cerebral salt wasting syndrome.

**Case presentation:**

A 70-year-old Korean man visited emergency room complaining of sudden onset vertigo and gait disturbance. Neurological exam showed left side ataxia, Horner syndrome, and right side hypesthesia. Brain magnetic resonance imaging disclosed acute infarction involving left lateral medulla. His neurological status was stabilized, but he began to complain of non-vertiginous dizziness and general weakness five days after admission. Serum sodium level dropped from 131 mEq/mL to 122 mEq/mL with reduced serum osmolarity of 265 mOsm/L. The diagnosis of syndrome of inappropriate secretion of antidiuretic hormone was made and we restricted fluid intake, but his symptoms worsened and his mental status became drowsy. Follow up serum sodium level was 108 mEq/L with volume loss, suggesting cerebral salt wasting syndrome. We treated him with hypertonic saline and his consciousness was recovered.

**Conclusion:**

This case shows symptomatic hyponatremia after lateral medullary infarction, providing insight about distinct pathogenesis of syndrome of inappropriate secretion of antidiuretic hormone and cerebral salt wasting syndrome.

## Background

Hyponatremia has been reported in patients with severe neurological diseases such as subarachnoid hemorrhage, head trauma and meningitis, and it is associated with high mortality [1]. The syndrome of inappropriate secretion of antidiuretic hormone (SIADH) and cerebral salt wasting syndrome (CSW) are the two main possible etiologies of hyponatremia due to a central nervous system (CNS) injury, yet the exact pathomechanism of them is still elusive [2]. It is of great interest to find the location that contributes to electrolyte disturbances after CNS injuries. Here we describe a lateral medullary infarction patient who experienced symptomatic hyponatremia with features of SIADH and CSW, and we discuss the possible pathomechanisms of these two conditions.

## Case presentation

**Figure 1:**
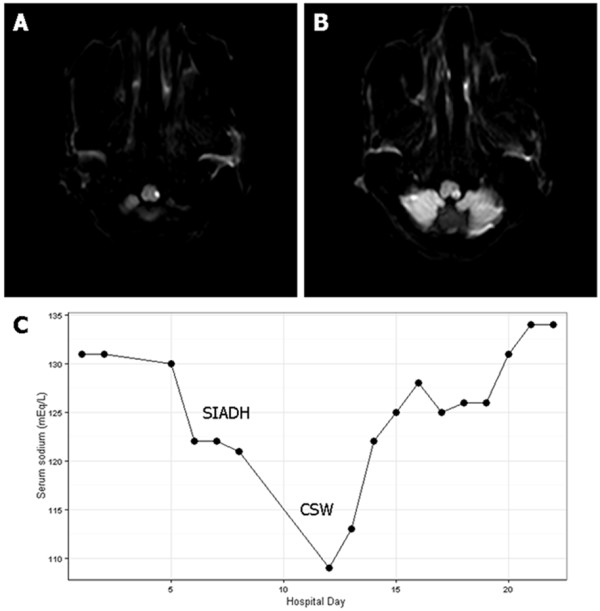
**Brain magnetic resonance imaging and serum sodium level.** Diffusion weighted image on admission showed left lateral medullary infarction **(A, B)** and serum sodium level after admission **(C)** demonstrated hyponatremia due to syndrome of inappropriate secretion of antidiuretic hormone (SIADH), and fluid restriction aggravated hyponatremia, suggesting cerebral salt wasting syndrome (CSW) as a final diagnosis.

A 70-year-old Korean man visited the emergency room complaining of the sudden onset of vertigo and gait disturbance. Neurologic examination showed left sided ataxia, Horner syndrome and right hypesthesia. Brain magnetic resonance imaging disclosed an acute infarct involving the left lateral medulla (Figure 1A, B). His previous medical history was unremarkable and he was a social drinker. He received oral aspirin 300 mg, atorvastatin 20 mg and intravenous hydration with normal saline 1 liter per day. Five days after admission, he began to complain of non-vertiginous dizziness and general weakness. A blood test on the 6^th^ day revealed a drop in the serum sodium level from 131 mEq/L on admission to 122 mEq/L, with reduced serum osmolarity of 265 mOsm/L (Figure 1C). The urine osmolarity was 844 mOsm/kg and urine sodium was 191 mEq/L. The patient was euvolemic and he was not taking any drugs except for aspirin and atorvastatin. He had normal thyroid and adrenal function. Under the impression that he had SIADH, we restricted the fluid intake thereafter. However his symptoms worsened with drowsy mentation and dehydrated volume status, and his body weight decreased from 50.0 kg to 46.1 kg. Follow up brain imaging did not reveal a new lesion, and the serum sodium level on the 12^th^ day was 108 mEq/L, with the urine sodium 58 mEq/L and the urine osmolarity 548 mOsm/kg. Considering the laboratory findings and the volume status, he was diagnosed with CSW rather than SIADH. We treated him with hypertonic saline and his mentation and dizziness improved, with a serum sodium level of 129 mEq/L.

## Conclusions

This case shows that a small brainstem lesion in the lateral medulla can cause hyponatremia that is severe enough to delay discharge from the hospital. Although the etiology of hyponatremia in this case is hard to ascertain, both SIADH and CSW should be considered after reviewing the patient’s volume status, electrolyte profiles and response to water restriction. This patient was diagnosed with SIADH because he was not dehydrated initially and urine was concentrated inappropriately, but later, the diagnosis was changed to CSW because he was not responsive to water restriction and volume loss was evident. The definition of CSW is renal loss of sodium during intracranial disorders leading to hyponatremia and a decreased extracellular fluid volume, whereas SIADH can be defined as hyponatremia with inappropriately concentrated urine and slightly increased intravascular volume due to excessive ADH [2,3]. The secretion of ADH is physiologically determined by osmotic and non-osmotic stimuli, and over-secretion of ADH from the posterior lobe of the pituitary gland is associated with SIADH after CNS disorders [2]. There has been a report of SIADH after lateral medullary infarction, and it was suggested that the nucleus tractus solitarius injury of the ventral medulla might cause failure of transmission of non-osmotic stimuli from the carotid sinus via the afferent vagal nerve, which causes disinhibition of ADH secretion at the pituitary gland, causing SIADH [4]. Several hypotheses exist to explain the pathophysiology of CSW after CNS disorders, including disrupted sympathetic tone which results in decreased renal sodium reabsorption and the failure to increase renin and aldosterone in hypovolemic conditions, and the inappropriate release of natriuretic factors [2]. Brain natriuretic peptide (BNP) released in the hypothalamus is a representative candidate for a CSW mediator, and physiological conditions known to increase its release include increased intravascular volume, distention of the left atrium, angiotensin II, and sympathetic stimuli [2].

The distinction between SIADH and CSW is often difficult because they have similar clinical and laboratory findings, but it is still important to distinguish between the two because the treatment strategies are different: SIADH patients should have restricted fluid intake, and CSW patients should receive salt supplements [5]. It is not certain why the patient's volume status, electrolyte profiles, and response to water restriction initially reflected SIADH and later CSW. The SIADH might have been due to disruption of the afferent vagal response via the nucleus tractus solitarius in the lateral medulla. The CSW could have been caused by the descending sympathetic tract injury which disrupted the sympathetic stimulus to the kidney and mild water retention due to initial intravenous hydration with normal saline. It is also conceivable to suspect that he initially experienced CSW without SIADH because he did not respond to water restriction.

### Consent

Written informed consent was obtained from the patient for publication of this case report and any accompanying images. A copy of the written consent is available for review by the Editor of this journal.

## Abbreviations

SIADH: Syndrome of inappropriate secretion of antidiuretic hormone; CSW: Cerebral salt wasting syndrome; BNP: Brain natriuretic peptide; CNS: Central nervous system.

## Competing interests

The authors declare that they have no competing interests.

## Authors’ contributions

JMK, contributed to manuscript writing and interpretation of the data. KYP contributed to manuscript drafting and critical revision of the manuscript for intellectual content. DHK, contributed to data interpretation and drafting of the manuscript. JHB, contributed to data acquisition. DU, contributed to acquisition and interpretation of the data. YCY, contributed to the critical revision of the manuscript for intellectual content. OSK, contributed to the critical revision of the manuscript for intellectual content. All authors read and approved the final manuscript.

## Pre-publication history

The pre-publication history for this paper can be accessed here:

http://www.biomedcentral.com/1471-2377/14/111/prepub
